# Mammal-Like Organization of the Avian Midbrain Central Gray and a Reappraisal of the Intercollicular Nucleus

**DOI:** 10.1371/journal.pone.0020720

**Published:** 2011-06-07

**Authors:** Marcy A. Kingsbury, Aubrey M. Kelly, Sara E. Schrock, James L. Goodson

**Affiliations:** Department of Biology, Indiana University, Bloomington, Indiana, United States of America; McGill University, Canada

## Abstract

In mammals, rostrocaudal columns of the midbrain periaqueductal gray (PAG) regulate diverse behavioral and physiological functions, including sexual and fight-or-flight behavior, but homologous columns have not been identified in non-mammalian species. In contrast to mammals, in which the PAG lies ventral to the superior colliculus and surrounds the cerebral aqueduct, birds exhibit a hypertrophied tectum that is displaced laterally, and thus the midbrain central gray (CG) extends mediolaterally rather than dorsoventrally as in mammals. We therefore hypothesized that the avian CG is organized much like a folded open PAG. To address this hypothesis, we conducted immunohistochemical comparisons of the midbrains of mice and finches, as well as Fos studies of aggressive dominance, subordinance, non-social defense and sexual behavior in territorial and gregarious finch species. We obtained excellent support for our predictions based on the folded open model of the PAG and further showed that birds possess functional and anatomical zones that form longitudinal columns similar to those in mammals. However, distinguishing characteristics of the dorsal/dorsolateral PAG, such as a dense peptidergic innervation, a longitudinal column of neuronal nitric oxide synthase neurons, and aggression-induced Fos responses, do not lie within the classical avian CG, but in the laterally adjacent intercollicular nucleus (ICo), suggesting that much of the ICo is homologous to the dorsal PAG.

## Introduction

The midbrain periaqueductal gray (PAG) comprises several histochemically and functionally distinct, semi-longitudinal columns that integrate descending information from limbic-hypothalamic forebrain areas and ascending sensory information from spinal and medullary afferents to coordinate downstream activation of motor processes that generate overt behavior [1], [2], [3], [4], [5]. The ventrolateral (VL) PAG regulates passive coping strategies and analgesia, whereas the dorsal PAG, which includes the dorsomedial (DM), dorsolateral (DL) and lateral (L) columns, regulates active coping strategies such as fight-or-flight behavior [1]. Importantly, different social stimuli reliably induce unique patterns of activation across the various columns. For instance, aggressive interactions produce Fos activation in the DL column of dominant male rodents [6], [7] and in the DM and L columns of subordinates [8], whereas predator exposure activates the DM and DL columns rostrally, plus L and VL columns caudally [8], [9], [10]. In contrast, copulation in male rodents activates only the most medial aspects of the DM and L columns in the rostral PAG [11].

To date, it remains unclear whether non-mammalian homologues of the PAG exhibit a similar functional organization. The large optic tectum of birds is laterally displaced and thus the central gray (CG) is stretched mediolaterally, rather than dorsoventrally as in mammals. This lateral extension of the avian CG has also been noted by Dubbeldam [12] who proposed that the CG and adjacent dorsomedial part of the intercollicular nucleus (ICo) share features with the mammalian PAG. Consistent with these ideas, we here hypothesize that the avian CG is organized like a folded open PAG, with medial CG being equivalent to ventral PAG and lateral CG/ICo being equivalent to dorsal PAG. To test this hypothesis, and to determine whether birds possess longitudinal columns that run rostrocaudally, we compared the immunocytochemical distributions of several neuropeptides and enzymes in mice and finches, as well as Fos activation patterns in birds (using the gregarious zebra finch, *Taeniopygia guttata*, and the territorial violet-eared waxbill, *Uraeginthus granatina*) with those known for rodents. We predicted that 1) the distinct cluster of neuronal nitric oxide synthase (nNOS) neurons that defines the DL column [13], [14] occupies the ventrolateral CG, 2) the dense innervation of the dorsal PAG by met-enkephalin (ENK) and substance P (SP) [5] lies laterally in the CG, 3) the vasoactive intestinal polypeptide (VIP) and tyrosine hydroxylase (TH) neurons that characterize the ventral third of the mammalian PAG [15], [16] occupy the medial third of the avian CG, and 4) dominance and defensive interactions would produce the strongest Fos activation in the lateral CG, whereas 5) copulation would most strongly activate the rostral CG, in a dorsal region adjacent to the ventricle.

The “folded open hypothesis” receives strong support from both the functional and immunocytochemical analyses. However, characteristic features of the dorsal PAG do not lie within the CG as classically defined, but in the adjacent ICo [17], a region known to be vocally active across a diversity of avian taxa [18].

## Results

### Immunohistochemical analyses of the avian CG

**Figure 1:**
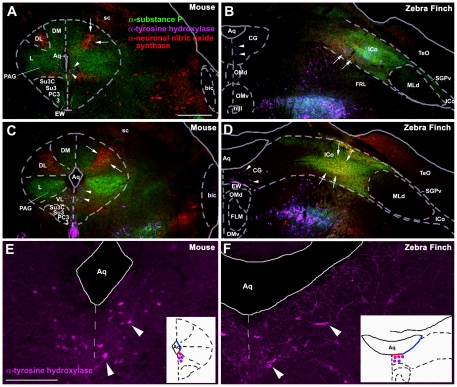
Immunocytochemical comparisons of the midbrains of mice and zebra finches. The PAG in mice (**A, C, E**) and the CG in finches (**B, D, F**) at rostral (A, B) and mid-rostral (C–F) levels of the midbrain, showing immunoreactive (-ir) cells and fibers for tyrosine hydroxylase (TH; purple), neuronal nitric oxide synthase (nNOS; red), and substance P (SP, green). Note that TH-ir cells are located ventrally along the aqueduct in mice (arrowheads in **A, C, E**) and medially along the aqueduct in finches (arrowheads in **B, D, F**) while SP-ir fibers and nNOS-ir cells are located in the lateral and dorsal columns of the PAG of mice (**A, C**) and in the lateral CG and ICo of finches (**B, D**). White arrows denote the cluster of small round nNOS-ir cells that is presumably homologous in mice and finches. TH-ir cells shown in C and D are shown at higher magnification in E and F, respectively. The schematic insets in E and F show the location of these TH-ir cells (purple dots) with respect to the aqueduct. While these neurons are located basally in both species, they are found in the ventral PAG of mice and the medial CG of finches (i.e. along red outline of aqueduct). Abbreviations for finches: Aq, aqueduct; CG, central gray; EW, Edinger-Westphal nucleus; FLM, medial longitudinal fasciculus; ICo, nucleus intercollicularis; MLd, nucleus mesencephalicus lateralis, pars dorsalis (auditory torus); nIII, oculomotor nerve; OMd/v, dorsal and ventral oculomotor nucleus; SGPv, stratum griseum periventriculare; TeO, tectum opticum. Abbreviations for mice: 3, oculomotor nucleus; bic, brachium inferior colliculus; DL, dorsolateral column of PAG; DM, dorsomedial column of PAG; L, lateral column of PAG; PAG, periaqueductal gray; PC3, parvicellular trigeminal nucleus; sc, superior colliculus; Su3, supraoculomotor central gray; Su3C, supraoculomotor cap; VL, ventrolateral column of PAG. Scale bar in A = 500 µm for A–D. Scale bar in E = 200 for E and F.

**Figure 2:**
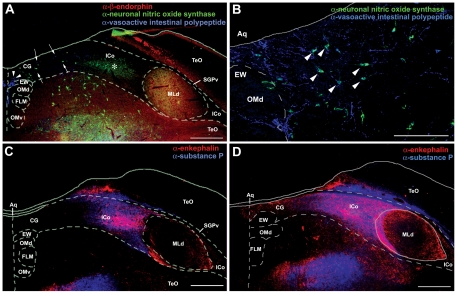
Chemoarchitecture of the avian CG/ICo. **A,** Immunoreactive label for β-endorphin (β-END; red), neuronal nitric oxide synthase (nNOS; green) and vasoactive intestinal polypeptide (VIP; blue) at a mid-rostral level of the midbrain. A distinct cluster of VIP-ir cells is observed in the medial CG (arrowheads) while a discrete cluster of nNOS-ir cells is found in the lateral CG/ICo (asterisk). Diffuse immunoreactive label for VIP and nNOS is present in the dorsomedial CG (arrows, see also B). β-END-ir fibers clearly define MLd at this midbrain level, yet diffuse β-END-ir fibers can be observed in lateral ICo at both rostral and mid-rostral levels (see Figure 5). **B,** A higher magnification of the VIP-ir cells and fibers and nNOS-ir cells in the dorsomedial CG that were highlighted by arrows in A. Many cells within this region are co-labeled by α-VIP and α-nNOS (white arrowheads). **C,** **D** Immunoreactive label for enkephalin (ENK; red) and substance P (SP; blue) in the lateral CG/ICo at a mid-rostral (C) and caudal (D) level of the midbrain. Note that at the caudal level, ENK-ir and SP-ir extend to the midline, with ENK-ir being more prominent. See Figure 1 for abbreviations. Scale bar in A, C and D = 500 µm. Scale bar in B = 200 µm.

Based on the lateral displacement of the avian midbrain, we predicted that histochemical features located ventrally in the mammalian PAG would be located medially in the avian CG, whereas histochemical features located dorsally in the PAG, would be located laterally in the CG. Immunohistochemical comparison of the midbrains of C57BL/6 mice and zebra finches strongly confirm these predictions. Whereas TH immunoreactive (-ir) cells are located basally along the aqueduct in both species, these neurons are present in the ventral PAG of mice (arrowheads in Figure 1A, C, E), while presumably homologous neurons are found in the medial CG of finches (arrowheads in Figure 1B, D, F). In both mice and birds, only light peptidergic immunoreactivity adjoins the region of the medial TH- and VIP-ir cell groups at mid-rostral levels, primarily VIP-ir cells and fibers that extend along the aqueduct and a few nNOS-ir cells (Figure 2A, arrows and Figure 2B).

**Figure 3:**
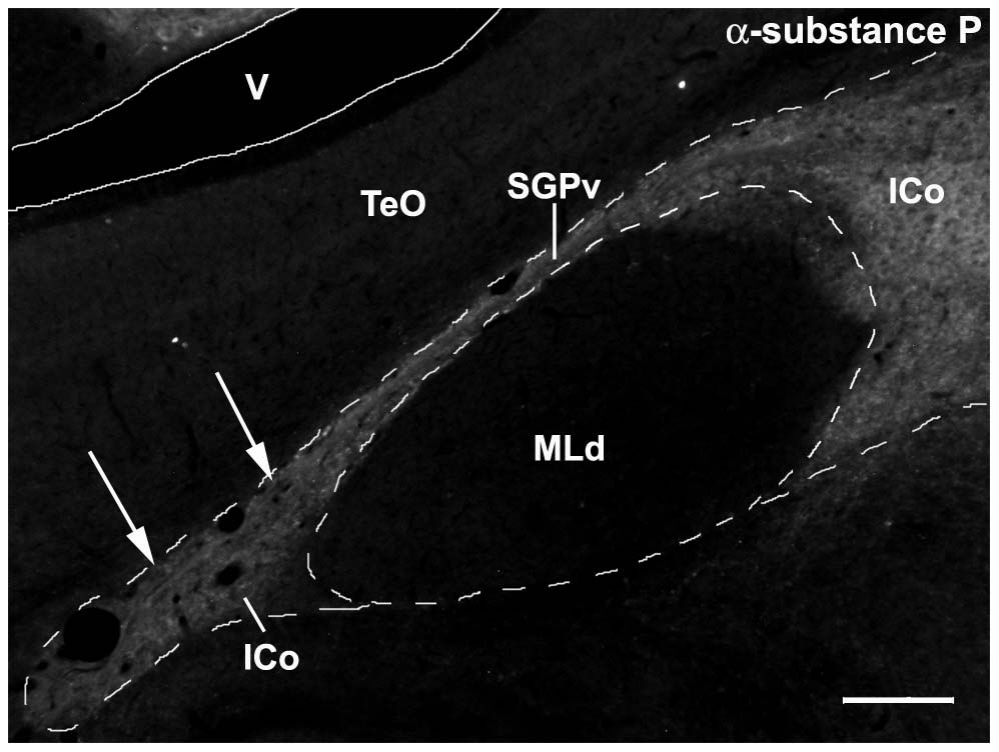
Immunoreactive label for substance P (SP) in the ICo surrounding MLd of a zebra finch at a mid-rostral level of the midbrain. Note that SP-ir fibers extend through the periventricular stratum (SGPv) into the ICo lateral to MLd (arrows) and appear to define this region. See Figure 1 for abbreviations. Scale bar = 200 µm.

**Figure 4:**
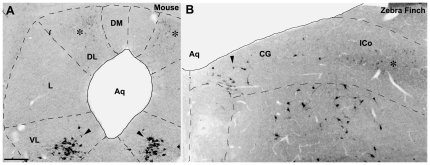
Immunoreactive label for neuronal nitric oxide synthase (nNOS) in the PAG of mice and CG/ICo of finches. **A, B,** A distinct population of large nNOS-ir cells is present in the VL column of the PAG (A, arrowheads) and the medial CG (B, arrowhead), in addition to the cluster of smaller nNOS-ir cells in the DL column of the PAG (A, asterisk) and lateral ICo (B, asterisk). See Figure 1 for abbreviations. Scale bar = 200 µm.

**Figure 5:**
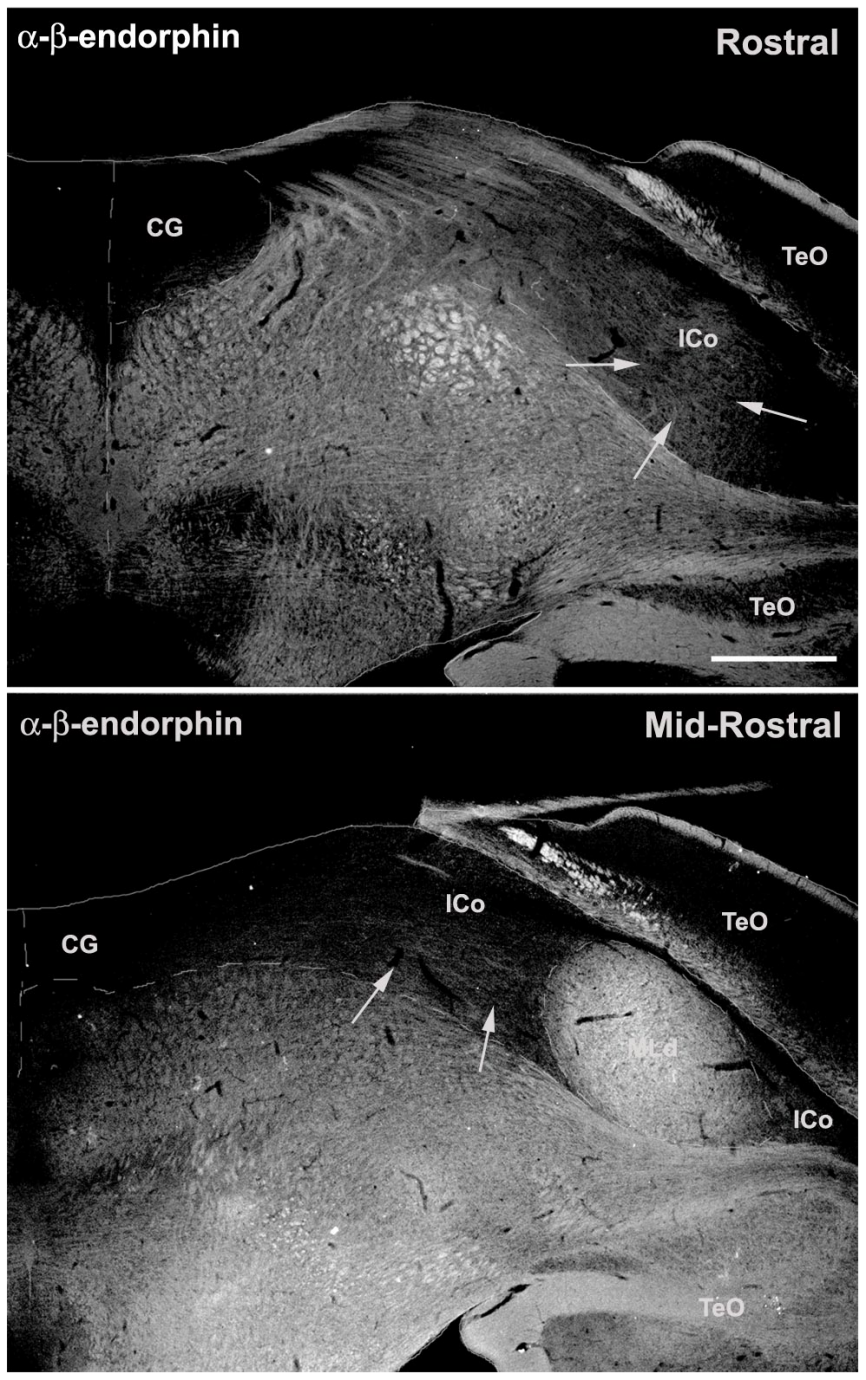
β-endorphin (β-END) in the midbrain of a male zebra finch. While β-END-ir fibers clearly define MLd at a mid-rostral level of the midbrain, diffuse β-END-ir fibers can be observed in the lateral ICo (arrows) at both rostral and mid-rostral levels. See Figure 1 for abbreviations. Scale bar = 500 µm.

In contrast, neuropeptides densely innervate the dorsal half of the PAG in mice and the lateral CG extending through the ICo in finches. In mice, SP-ir fibers innervate the DM and L columns (the latter much more heavily) of the rostral and mid-rostral PAG (Figure 1A, C), whereas a comparable SP innervation is observed for the lateral CG and ICo in finches, also at rostral and mid-rostral levels (Figure 1B, D). Furthermore, just as the apex of the DM column exhibits light SP-ir fibers (Figure 1A, C), so does the ICo region lateral to the nucleus mesencephalicus lateralis, pars dorsalis (MLd; Figure 3). ENK-ir fibers heavily overlap the SP-ir fibers of the lateral CG/ICo at mid-rostral levels in finches (Figure 2C), and a similar overlap is observed in the dorsal PAG of mice at mid-rostral levels, in agreement with the overlap of ENK and SP mRNA in the dorsal PAG of rats (note that immunodetection of these peptidergic cells are likely not possible without colchicine pretreatment) [5]. At caudal levels of the avian CG, ENK-ir and SP-ir fibers overlap and extend medially towards the midline, although the ENK-ir labeling is more prominent (Figure 2D) and a comparable descending ENK-ir innervation of the VL column is observed in mice (cf. [16], [19]). In addition, the distinct column of small round nNOS-ir cells that characterizes the DL column in mice (average soma size, 9.9 µm+/−0.567 µm; Figure 1A, C, arrows) is observed as a distinct cluster of small round nNOS-ir cells in the ventrolateral CG/ICo region in finches (average soma size, 10.2 µm+/−0.318 µm; arrows in Figure 1B, D, see also asterisk in Figure 2A and Figure 4B) within territory classically defined as the ICo [17]. The large nNOS-ir cells clustered within the ventral PAG more caudally in mice (average soma size, 18.3 µm+/−0.978 µm; arrowheads in Figure 4A) likely correspond to the large nNOS-ir cells observed in the medial CG of finches (average soma size, 18.4 µm+/−0.652 µm; arrowhead in Figure 4B). The pattern of labeling for β-endorphin (β-END) is particularly interesting, since β-END-ir fibers below the CG/ICo appear to delineate the ventral boundary of this area very well, and also distinguish MLd from the surrounding ICo (Figure 2A). However, diffuse β-END-ir fibers can be observed in the lateral ICo at rostral and mid-rostral levels (Figure 5, arrows).

### Functional columns of the avian CG

**Figure 6:**
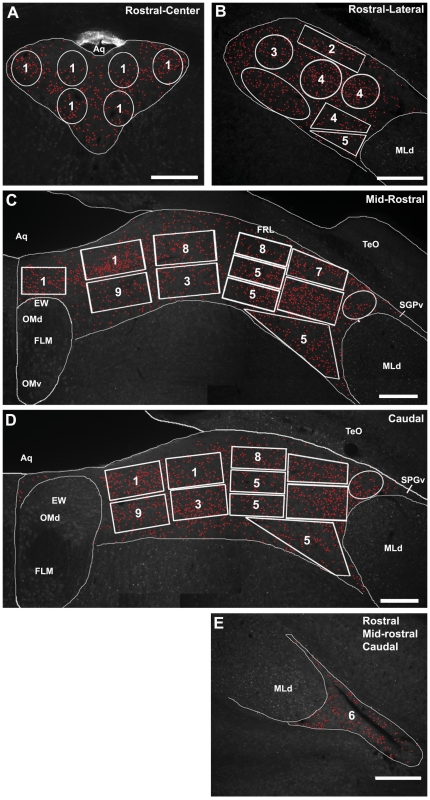
An exemplar set of photomicrographs from the CG and ICo of a male zebra finch, showing the gridwork of boxes and polygons that were used for counts of Fos-ir cells at each of the three levels analyzed in zebra finches and violet-eared waxbills. Fos-positive cells were counted in a separate Photoshop layer using the paintbrush tool (red dots). **A, B** Boxes and polygons used to examine Fos activation at a rostral midbrain level in the medial CG (**A**) and lateral CG/ICo (**B**). **C, D** Boxes and polygons used to examine Fos activation at mid-rostral (**C**) and caudal (**D**) midbrain levels. **E,** The triangular area of ICo lateral to MLd. This area was analyzed for each of the 3 rostrocaudal levels. Given the large individual variability in the size and shape of this area, we traced the entire triangular ICo and conducted cell counts within the outline. As with cell counts from the boxes and polygons, all Fos-ir cell counts were standardized to a unit of Fos-ir cells per 100 µm^2^. Based on similar response properties in contiguous sampling areas, including those that are rostrocaudally contiguous, the 36 separate sampling areas (per side) were reduced to 9 functional zones (see methods and Fig. 8A). Note that in B, C, D and E, only the left midbrain is shown, yet we analyzed both a left and right midbrain section for each animal at each rostrocaudal level. Note also that at the most rostral level analyzed, we present both the left and right medial CG in A. See Figure 1 for abbreviations. Scale bars = 250 µm.

**Figure 7:**
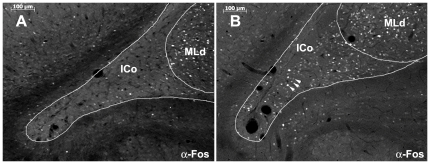
Enhanced Fos activation in the ICo region lateral to MLd (zone 6 in **Figure 8** A, D). Greater Fos activation is observed in a dominant violet-eared waxbill following a resident intruder encounter (**B**) as compared to a waxbill in the control condition (**A**). The white arrowheads in B illustrate examples of Fos-ir cells with different levels of Fos protein in the nucleus.

**Figure 8:**
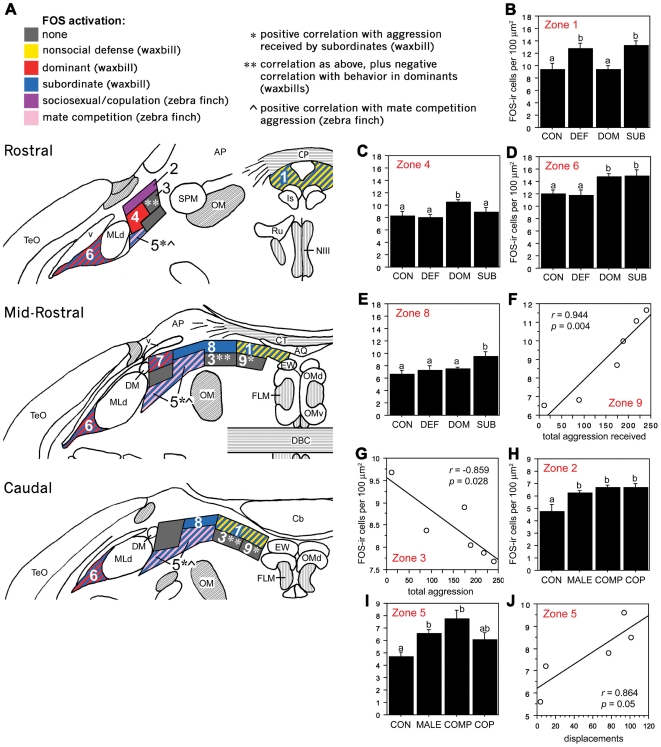
Functional columns of the avian CG/ICo as delineated by Fos induction. **A,** Schematic rostrocaudal representation of Fos responses in the avian CG and ICo following exposure to different social conditions (see legend). **B–E,** Fos-ir cell counts in male violet-eared waxbills exposed to a handled control condition (CON), nonsocial defense manipulation (pursuit by a human hand; DEF) and a resident-intruder encounter (dominant, DOM, and subordinate, SUB) within zones 1, 4, 6 and 8, respectively. **F,** Fos-ir cell counts in zone 9 of subordinate animals are positively correlated with the total number of aggressive behaviors received. Similar results are obtained for zones 3 and 5 (see schematic). **G,** Fos-ir cell counts in zone 3 of dominant animals are negatively correlated with total number of aggressive behaviors displayed. **H–I,** Fos-ir cell counts of male zebra finches following exposure to a handled control condition (CON), a conspecific male (MALE), a mate competition interaction (COMP; includes both courtship and aggression) and a copulatory interaction with a female (COP). **J,** Fos activation in zone 5 is positively correlated with the number of displacements the dominant subject directed at the competing male during mate competition. Data in panels **B–E, H, I** are shown as means +/− SEM. Different letters above the error bars denote significant group differences (Fisher PLSD p<0.05 following significant ANOVA). Abbreviations: AP, area pretectalis; Cb, cerebellum; CT, commissura tectalis; CP, commissura posterior; DBC, decussatio brachiorum; DM, dorsomedial nucleus; Is, nucleus interstitialis; OM, occipitomesencephalic tract; Ru, nucleus ruber; SPM, nucleus spiriformis medialis. See Figure 1 for other abbreviations.

Analyses of Fos responses in the 39 sampling areas (Figure 6) revealed 9 functional zones of the CG and the ICo that are differentially responsive to the various social conditions (see Methods for details and Figure 7 for an example), and which mostly form longitudinal columns that span multiple rostrocaudal levels (Figure 8). Note that our delineation of functional zones based on Fos response profiles was conducted without making *a priori* assumptions about rostrocaudal contiguity. To maintain a high degree of resolution, we assigned different zone numbers to rostrocaudally continguous areas that showed only minor differences in response profile. Most notable in this regard is the assignment of different zone numbers to zone 4 (level 1) and zone 7 (level 2). Both of these areas exhibited a Fos response in dominant waxbills whereas only zone 7 exhibited a Fos response in subordinate individuals (see below for details). Other zones showed greater consistency across levels. A schematic of these zones, color-coded by response profile, is presented in Figure 8A. In general, the functional organization of the avian CG is strongly consistent with our predictions (see Discussion).

#### Nonsocial defense (waxbills)

Relative to control and dominant subjects, waxbills in the nonsocial defense group exhibited significantly greater Fos induction in a midline column that spanned all three rostrocaudal levels (zone 1, *F*_(3,20)_ = 6.929, *p* = 0.0022; Figure 8A, B). A similar response was observed in subordinate subjects, as described below. This is the only column in which a significant Fos response was observed for the nonsocial defense group.

#### Dominance (waxbills)

Dominant male waxbills exhibited Fos responses that were restricted to the far lateral portions of our sampling area, including a zone on the medial edge of MLd, which exhibited highly selective responses in the dominant subjects, although this was observed only at the rostral-most level (zone 4, *F*_(3,20)_ = 2.919, *p* = 0.0503; Figure 8A, C). Just caudally, at the mid-rostral level, a topographically similar and contiguous area showed a significant Fos response in both the dominant and subordinate groups (zone 7, *F*_(3,20)_ = 5.554, *p* = 0.0061; Figure 8A). A virtually identical response pattern was observed for the lateral-most ICo (i.e., lateral to MLd; zone 6, *F*_(3,20)_ = 4.531, *p* = 0.0140; Figure 8A, D, see also Figure 7). Zone 6 formed a functional longitudinal column that spanned the full rostrocaudal extent of the ICo.

#### Subordinance (waxbills)

Subordinate waxbills exhibited widespread activation across the CG and ICo, with the notable exception of the ventromedial portions of the sampling area (gray boxes in Figure 8A). In addition to the zones mentioned above, in which subordinates exhibited activation that overlapped with other groups, the subordinate waxbill group exhibited significant activation of the dorsocentral CG/ICo (zone 8, F_(3,20)_ = 3.275, p = 0.0424; Figure 8A, E), and a contiguous area that extended ventrolaterally to MLd (zone 5, *F*_(3,20)_ = 2.993, *p* = 0.050; Figure 8A). The number of Fos-ir cells in zone 5 correlates positively with the number of aggressive behaviors received by subordinate subjects (*r* = 0.899, *p* = 0.0149) and is also activated by mate competition aggression in zebra finches (see below). The ventromedial areas that showed no group differences (zones 3 and 9) nonetheless exhibit Fos-ir cell counts that are also positively correlated with aggression received by subordinates (zone 3, *r* = 0.830, *p* = 0.0362; zone 9, *r* = 0.944, *p* = 0.0046; Figure 8F). We have presented these two sampling areas as different zones because zone 3 also exhibits a negative correlation between Fos-ir cell counts and the aggression displayed by the dominant subjects (*r* = −0.859, *p* = 0.0282; Figure 8G). Notably, the response profiles of zones 3, 5, 8 and 9 are maintained across rostrocaudal levels, and hence appear to characterize robust and functionally distinct longitudinal columns.

#### Mate competition and copulation (zebra finches)

Significant Fos responses to copulation were restricted to a rostral, dorsolateral region of the ICo directly adjacent to the ventricle (zone 2; Figure 8A, H; *F*_(3,15)_ = 7.199, *p* = 0.0032). This area also exhibited significant responses to a same-sex conspecific, which is likely an affiliative context for zebra finches, and mate competition, a context that elicits a combination of both courtship and aggression. Fos induction was very similar in the two groups exposed to females (mate competition and copulation). Mate competition in resident males also produced a strong response in the ventrolateral ICo, adjacent to the medial edge of MLd (zone 5; Figure 8A, I; *F*_(3,15)_ = 5.552, *p* = 0.0093), and Fos-ir cell counts in this area correlated positively with the number of displacements the resident male directed at the intruder male (Figure 8J; *r* = 0.864, *p* = 0.05). Notably, this positive correlation in zone 5 is similar to that observed for aggression received in subordinate waxbills, and no significant correlation was observed in this area for dominant male waxbills. Although counterintuitive, this result is consistent with previous studies showing that aggression in mate competition contexts is regulated in a manner very differently from aggression in other contexts [20], [21]. Mate competition further induced a significant Fos response within a small region on the dorsomedial margin of MLd (*F*_(3,15)_ = 5.112, *p* = 0.0124), which likely corresponds to the dorsal medial nucleus classically assigned to ICo [22] and at least in part to the intercollicular core nucleus as defined in the chick ([23], [24]; see Discussion).

## Discussion

Based on the lateral displacement of the optic tectum in birds, we hypothesized that the avian CG is organized much like a folded open mammalian PAG, with the medial CG being equivalent to the ventral PAG and the lateral CG being equivalent to the dorsal PAG (which includes the DM, DL and L columns). Although this hypothesis receives strong support from our data, the predicted pattern of results is observed *only* if the avian CG is expanded to include virtually all of the ICo as presently defined. Thus, based on histochemistry, position relative to other structures, Fos responses to social stimuli, and the presence of functionally distinct longitudinal columns, we propose that the avian CG and ICo are collectively homologous to the mammalian PAG, and that the ICo is specifically homologous to the dorsal PAG (i.e. DM, DL and L columns).

### Chemoarchitectural analyses support a folded open model of the avian CG/ICo

Our comparisons of histochemistry in birds and mammals were of two kinds: 1) Direct comparisons of immunolabeling for TH, SP, nNOS and ENK in the midbrains of mice and finches, and 2) comparisons of β-END and VIP immunoreactivity in zebra finches with the patterns reported in the mammalian literature. In all cases, these comparisons support our predictions. Several histochemical features of the VL PAG, such as the TH- and VIP-ir cells that lie ventrally along the cerebral aqueduct in mammals [5], [16], are located medially along the aqueduct in finches, and at mid-rostral levels, peptidergic innervation of the VL PAG and medial CG is comparably light. Similarly, birds and mice exhibit presumably homologous large nNOS-ir cells within the caudal midbrain, which lie medially in the CG and ventrally in the PAG (Figure 4, arrowheads).

Likewise, markers that characterize the dorsal PAG in mammals were observed in the lateral CG/ICo of birds. For instance, a dense plexus of SP-ir fibers is localized to the DM and L columns in the rostral and mid-rostral PAG of mice, and in the lateral CG/ICo of finches at comparable rostrocaudal levels. A similar pattern is observed for ENK. In mammals, ENK-ir fibers and ENK mRNA are predominately localized to the L column, and to a lesser extent in the DM column of the dorsal PAG, whereas ENK labeling in the VL column is observed more caudally [5], [19]. In the current study, robust ENK-ir in zebra finches was observed in the lateral CG/ICo at all rostrocaudal levels while a thin band of ENK-ir extended medially from ICo to the midline at caudal levels. This latter pattern is similar to the ENK-ir pattern in the VL column of mice [19]. Furthermore, the overlap of ENK mRNA and SP mRNA in the L column of the dorsal PAG of rats [5] is also observed with ENK-ir and SP-ir fibers in the lateral CG/ICo of zebra finches, and in the L column of mice. Perhaps the most striking evidence for a correlation between the dorsal PAG and the lateral CG/ICo is the finding that the distinct group of nNOS cells that characterize the DL column in mice and rats ([13], [14]; Figure 1A, C and Figure 4A) is found as a distinct cell cluster in the ventrolateral CG/ICo (Figure 1B, D and Figure 4B). Finally, β-END-ir fibers were observed in the lateral ICo at rostral and mid-rostral levels (Figure 5), consistent with β-END-ir label in the dorsal PAG of cats that spans the DM, DL and L columns [25].

**Figure 9:**
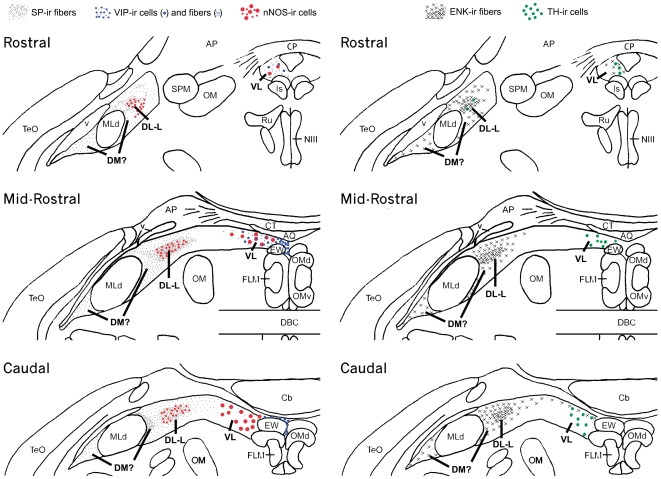
A summary schematic illustrating the organization of various neuropeptides and neurotransmitters within the zebra finch CG and ICo across multiple rostrocaudal levels that suggest the existence of longitudinal columns based on immunohistochemistry. The distribution of substance P (SP), vasoactive intestinal polypeptide (VIP) and neuronal nitric oxide synthase (nNOS) are shown at three rostrocaudal midbrain levels on the left while enkephalin (ENK) and tyrosine hydroxylase (TH) are shown on the right. A legend is shown at the top. The density of dots for SP-ir and x's for ENK-ir correspond to the intensity of immunoreactive fibers. Areas of the avian CG and ICo that are hypothesized to correspond to specific PAG columns in mammals are indicated in bold type at each rostrocaudal level. Note that for each neuropeptide or neuromodulator examined, each is found in a specific mediolateral position that is comparable across the different rostrocaudal levels, suggesting a longitudinal organization of neuropeptides and neuromodulators within the avian midbrain.

In summary, whereas some histochemical comparisons indicate a fairly specific correspondence between columns of the mammalian PAG and CG/ICo (e.g., as shown for the medial CG and VL PAG, both of which express large VIP- and nNOS-ir cells), other histochemical features suggest a less specific correspondence. For instance, just as the peptidergic innervations of the DL, L and DM columns are poorly differentiated in mice, so too are they poorly differentiated in the lateral CG/ICo of finches, which allows us only to state with confidence that the lateral CG/ICo corresponds to the dorsal PAG in mice, which encompasses the DM, DL and L columns (Figure 9).

### Immediate early gene analyses support a folded open model of the avian CG/ICo

Aggressive interactions induce robust Fos activation of the dorsal PAG in rodents [6], [26], although subordinate rodents tend to exhibit substantially more Fos induction than do dominant males [8]. Similarly, and consistent with our model, dominant zebra finches exhibit activation of the lateral ICo following mate competition, and only the lateral-most ICo (i.e. adjacent to MLd) exhibits Fos induction in both dominant and subordinate waxbills, similar to the dorsal PAG of mammals. Activation in subordinate violet-eared waxbills covers a broader portion of the lateral ICo than that observed for dominant males, and for much of this territory, subordinates exhibit greater activation than do all other groups, including the nonsocial defense group. This highlights one modest inconsistency with rodents, in that nonsocial defense in waxbills (escape from pursuit by a human hand), activates only the most medial CG/ICo region, whereas predator exposure in rats activates not only the VL, but also the DM/DL columns [8], [9]. However, this may simply reflect a difference in the nature of the fear stimulus and/or the coping or recuperative responses of the test subjects. Fos activation of the VL PAG is associated with recuperative responses following intense physical activity and/or emotional stress [27], which may also explain why waxbills in both the subordinate and nonsocial defense groups exhibited equivalent Fos responses in the medial CG.

Copulation in male rats activates dorsomedial and lateral regions directly adjacent to the aqueduct of the rostral PAG [11], and consistent with our model, copulation activates the rostral ICo in male Japanese quail [28], [29]. Copulation also induces Fos within a dorsal, periventricular portion of the rostral ICo in zebra finches, although this area responds to other sociosexual stimuli as well, perhaps reflecting the highly gregarious nature of zebra finches and subject isolation prior to experiments.

The magnitude of Fos activation following exposure to aggression, sex or fearful stimuli is remarkably high in mammals [9], [26], and much more striking than that observed in the present experiments. However, whereas very low levels of constitutive Fos activity are observed in the mammalian PAG [9], [26], songbirds exhibit exceptionally high levels of constitutive Fos expression in the CG and many other parts of the brain [30], [31].

### Redefining ICo as part of the avian CG

ICo has traditionally been considered a vocalization area based on early studies in a variety of bird species, which showed that electrical stimulation of the midbrain, and ICo in particular, can elicit natural vocalizations [18], [32], [33], [34], [35], [36], [37]. Vocalizations have also been elicited from the peripheral region of the torus semicircularis in lizards [38] and tegmental territory immediately medial to the torus semicircularis in fish [39], [40], which is considered the piscine CG based on position and interconnections with other brain areas [40]. Many mammals also exhibit vocalizations that resemble natural calls following electrical stimulation of the midbrain PAG ([41], rat; [42], bat; [43], cat; and [44], monkey; see [45] for a review in mammals). In fact, the hypothesis that the PAG of mammals may be homologous to the ICo of birds was suggested as early as 1965 by Brown when he proposed that the avian torus semicircularis corresponded to the mammalian PAG based on the location of midbrain vocalization centers across different species [32].

The idea that vocal regulation in birds is tightly integrated with other midbrain behavioral processes was proposed by Seller [18] after studies showed that stimulation of the ICo region medial to MLd evoked fear responses and components of threat displays, in addition to natural calls [33], [34], [36], [46]. Further evidence that the ICo is involved in complex behavior beyond vocalization was provided by studies in Japanese quail in which males that emitted few crows but were allowed to copulate with females showed higher immediate early gene activation in rostral ICo compared to males that crowed frequently within an empty arena [28]. Similarly, self-stimulation of the midbrain PAG in humans has been shown to induce complex emotional states [47], [48] in addition to eliciting natural vocalizations [48].

Notably, based on histochemical and developmental considerations, Puelles and colleagues recently proposed a laterally expansive PAG in the chick, but these authors still recognize a distinct ICo (or at least its “core nucleus”), which they define primarily on the basis of vocal activity [24]. As just noted, however, the CG/PAG of other vertebrate taxa represent the major vocal conduit in the midbrain, and although adjacent tegmentum is also sometimes vocally active in mammals, it lacks the heavy peptidergic innervation that characterizes the mammalian dorsal PAG, the ICo and the intercollicular core nucleus of Puelles et al. [23], [24].

Connectional studies also demonstrate that features of the avian ICo are consistent with those of the dorsal PAG. In mammals, overlapping projections from the spinal cord, dorsal column nuclei and sensorimotor cortex define an intercollicular region that lies substantially within the lateral portion of the dorsal PAG, medial to the inferior colliculus [49], [50], [51], [52], [53]. Interestingly, the dorsal column nuclei, spinal cord and hyperstriatum accessorium (HA) of the rostral Wulst (an area thought to be homologous to the mammalian somatosensory cortex; [54], [55]) all project onto the avian ICo territory adjacent to MLd [56], [57], [58]. Furthermore, projections of the ventromedial nucleus of the hypothalamus, which heavily target and clearly define the dorsal/dorsolateral PAG in mammals [59], target virtually the entire classical ICo of birds, rather than the classical CG, and strongly differentiate this area from surrounding tegmentum [60].

Thus, the anatomical studies mentioned above, together with the functional and chemoarchitectural data presented here, suggest that much of what has been classically defined as the “ICo” in birds is, in fact, homologous to columns of the dorsal PAG of mammals. The idea that the ICo and CG share characteristics with the PAG was suggested by Dubbeldam and den Boer-Visser following an analysis of midbrain connectivity and distribution of neuromodulators [12]. Furthermore, Dubbeldam [61] suggested that the dorsomedial nucleus of ICo is functionally equivalent to the lateral parts of the PAG based on respiratory pathways in birds and mammals (see below for further details). In the present study, we attempted to compare all regions of the CG and ICo with specific PAG columns, as well as determine whether the avian CG/ICo contains functional longitudinal columns. Based on our results, together with previous literature, CG and ICo regions that are homologous to several of the PAG columns appear clear. For instance, immunolabeling for TH and nNOS defines the VL (TH and nNOS) and DL (nNOS) columns in mice and the medial CG and lateral ICo, respectively, in birds (Figure 1 and Figure 4). Furthermore, the L column in mammals likely corresponds to an area in the lateral ICo of birds that is medial to the boundary with MLd, and is delineated by intense SP immunolabeling (Figure 1). Interestingly, this intense SP labeling in lateral ICo overlaps the nNOS labelling at all rostrocaudal levels (Figure 9), suggesting that the avian homologues of the DL and L PAG columns overlap (Figure 1D), in contrast to what is observed for mammals (Figure 1C). Further evidence that the avian homologue of the L PAG column lies within the ICo region just medial to MLd is provided by studies examining midbrain projections to nucleus retroambiguus (NRA), a group of premotor neurons in the caudal medulla that are involved in vocalization via their control of expiration and laryngeal musculature [62], [63], [64]. In mammals, projections to NRA arise predominantly from the L PAG column [65], [66]. Consistent with our predictions, projections to the NRA in zebra finches arises from the dorsomedial nucleus of the ICo, a region directly medial to MLd (Figure 4B in [22]) that is known to be a prominent midbrain vocal control center in songbirds [67]. An outstanding question is the location of the avian homologue of the DM column of the PAG. Based on the folded open model of the PAG, we predict that the DM homologue is located lateral to the distinct nNOS and intense SP labeling in ICo and potentially extends to the region of ICo lateral to MLd. While none of the immunohistochemical markers used in the present study marked only the DM column, this column contains lighter SP-ir that than found in L, as does the ICo area immediately surroundly MLd (both medial and lateral to MLd; Figure 1D and Figure 3). Furthermore, significant Fos activation is observed in the dorsal PAG of resident male hamsters and rats exposed to an intruder [6], [26] and a similar Fos activation was observed in the ICo regions directly adjacent to MLd (red in Figure 8) of male waxbills exposed to a resident intruder paradigm, including the lateral-most ICo. Thus, we hypothesize that the region lateral to MLd is part of the dorsal PAG and that the homologue to the DM column lies within this region. In summary, our data support the claim that the VL PAG and CG are likely homologous, as well as the DL/L PAG and the ICo region medial to the MLd. However, further evidence is needed to more clearly specify the boundaries of the avian homologue of the DM column.

## Materials and Methods

### Ethics Statement

Experiments were performed in accordance with federal and institutional guidelines for the ethical treatment of animals. Tissue for the present studies was collected under protocol S00032 that was approved by the Institutional Animal Care and Use Committee at the University of California, San Diego, and protocols 07-026, 07-027 and 09-035 that were approved by the Bloomington Institutional Animal Care and Use Committee at Indiana University.

### Animals and housing

#### Chemoarchitecture studies

Tissue for double- and triple-label chemoarchitecture studies was collected from 3 male C57BL/6 mice and 4 male zebra finches, each of which yielded three series for labeling. In addition, we utilized a large amount of material that was generated as part of previous experiments, including TH immunolabeling in 91 zebra finches (82 male, 9 female; [68]) and 19 violet-eared waxbills (10 male and 9 female; [69]). Fos studies: Twenty-eight adult male zebra finches and twenty-four adult male violet-eared waxbills were used for the present behavioral experiments. Zebra finch males were housed with same-sex conspecifics in 61 cm W×36 cm D×41 cm H wire cages while violet-eared waxbills were individually housed in 61×36×41 cm wire cages. Animals were maintained on a 14L∶10D photoperiod and provided seed mix and water *ad libitum*.

### Immunocytochemical analysis of zebra finch and C57/BL/6 mouse brains

Three series of 40 µm coronal tissue sections were collected from 3 male C57/BL/6 mice and 4 male zebra finches. These were immunofluorescently labeled according to previously published protocols [68], [70]. For mouse tissue, we used various combinations of the following primary antibodies: sheep anti-TH (Novus Biologicals, Littleton, CO), monoclonal mouse anti-nNOS (Sigma-Aldrich, St. Louis, MO), rabbit anti-SP (Chemicon, Temecula, CA), and monoclonal mouse anti-ENK (Chemicon). Primary antibodies were visualized with secondary antibodies raised in donkey and conjugated to Alexa Fluor 488, 594 and 680 (Invitrogen, Eugene, OR). We additionally referenced tissue from a previous study [71] that was labeled using an AVP antibody raised in guinea pig (Bachem, Torrance, CA), and tissue from alternate series of the same brains that we labeled for nNOS as just described. For zebra finch tissue, we used various combinations of the TH, nNOS, ENK and SP antibodies listed above, plus guinea pig anti-β-END (Bachem), and both rabbit and guinea pig anti-VIP (Bachem). We have not previously employed the nNOS antibody in finches; however, deletion of the primary antibody yields no labeling and the pattern of labeling with this antibody closely matches the distribution of nNOS elements in rodents (e.g., [13], [14]; also present data) and the distribution of nicotinamide adenine dinucleotide phosphate-diaphorase elements in birds (e.g., [72]). Primary antibodies were labeled using secondary antibodies conjugated to either Alexa Fluors (488, 594 or 680) or biotin, which was subsequently visualized using streptavidin conjugated to Alexa Fluors (see [70], [73]). Photomicrographs shown here were shot at 5× magnification using a Zeiss Axioskop microscope (Carl Zeiss, Jena, Germany) and an Optronics Magnafire digital camera (Optronics, Galeta, CA) linked to an Apple Macintosh MacPro computer (Apple Corporation, Cupertino, CA). Image color, contrast and brightness level were adjusted in Photoshop CS3 (Adobe Systems, Seattle, WA).

### Behavioral testing in violet-eared waxbills

Male violet-eared waxbills were acclimated to a walk-in sound isolation booth by placing their home cages into the booth for 2 hrs per day for 2 days. Prior to behavioral testing, waxbills remained in the booth overnight. Both resident and intruder subjects were placed in the same booth, but were visually isolated. The following morning, subjects were exposed to one of four experimental manipulations in a 7 min test: a control condition in which no stimulus bird was introduced (*n* = 6); a nonsocial defense manipulation in which the subject was pursued by a human hand 40 times, distributed across the 7 min (*n* = 6); or a resident-intruder encounter in which the subject was either a dominant resident (*n* = 6) or a subordinate intruder (*n* = 6). For each condition, lights were turned off, food was removed, and to control for handling of the intruders, all subjects were caught with the aid of a flashlight, held for 3 sec, then placed on the cage floor. The lights were turned on and behavior was observed for 7 min using a curtain blind. We recorded displacements, threats, pecks and agonistic calls. At the end of testing, lights were turned off, subjects were again handled, and intruder animals were returned to their home cages. Lights remained off until perfusion 90 min after the start of testing. Lights were left off in order to prevent agonistic vocal interactions between the two subjects in the sound booth, which would produce Fos responses to behavioral interactions outside of the 7 min period of behavioral quantification. Lights were not left off in the next experiment, since subjects were housed alone. Given that subjects in both experiments were devoid of social stimulation after the behavioral test, this small point of difference between studies is not expected to impact socially-induced Fos response profiles; regardless, no statistical comparisons are made between the two datasets.

### Behavioral testing in zebra finches

The behavioral testing in male zebra finches has been fully described [68] and is briefly summarized as follows: The day before behavioral testing, zebra finches were removed from same-sex housing and placed into individual 36 cm W×33 cm D×17 cm H test cages in a quite room. The following morning, subjects were exposed to one of four experimental conditions, as described below. For each condition, lights were turned off, food was removed and the stimulus bird(s) was placed in the subject's cage. The lights were turned on and behavior was recorded for 10 min. After testing, the lights were then turned off, the stimulus bird(s) was removed, food was reintroduced, lights were turned on and the subject was left in the cage until 90 min after the start of the test. Birds were then perfused for immunocytochemical labeling of Fos protein. The four conditions were: 1) a control condition in which no stimulus bird was introduced (*n* = 4); 2) exposure to a conspecific male (*n* = 5); 3) a mate competition paradigm [74] in which the subject was exposed to both a conspecific male and female (*n* = 5); and 4) sexual interactions with a female. Only males that successfully mounted the female (*n* = 5) were included.

### Immunocytochemistry and image analysis for behavioral experiments

Tissue from zebra finches and violet-eared waxbills was processed and immunofluorescently double-labeled for TH and Fos as previously described [68], using a rabbit anti-Fos antibody (Santa Cruz Biotechnology, Santa Cruz, CA), a sheep anti-TH antibody (Novus Biologicals) and donkey anti-rabbit and anti-sheep secondary antibodies conjugated to Alexa Fluor 594 and 488, respectively (Invitrogen).

Monochrome photomicrographs were shot at 10× magnification at three different rostrocaudal levels of the midbrain for each animal (corresponding approximately to plates P 0.2, P 0.8 and P 1.0 of the canary atlas; [75]) using a Zeiss Axioskop microscope (Carl Zeiss) and an Optronics Magnafire digital camera (Optronics) linked to an Apple Macintosh MacPro computer (Apple Corporation).

A montage for each rostrocaudal level was created in Adobe Photoshop 7 (Adobe Systems) using consecutive monochromes images captured from the most medial aspect of the CG through the most lateral aspect of the ICo just beyond MLd, homologue of the inferior colliculus. Fos cell counts were conducted from montaged images in Photoshop using a gridwork of boxes and polygons that spanned the entire mediolateral extent of the CG and ICo for each level (Figure 6). Montages of both the left and right midbrain at each rostrocaudal level were sampled and counted for each animal. Our goal in this process was to generate a reasonable level of spatial resolution without oversampling. The actual size and shape of these boxes and polygons was largely dictated by the shape of the CG and the ICo, and individual variation in those parameters. The number of boxes and polygons per side (i.e. right versus left) was 10 (3 for rostral-center and 7 for rostral-lateral), 12 (mid-rostral) and 11 (caudal) for the three rostrocaudal levels. An additional area lateral to MLd corresponding to the triangular portion of the lateral-most ICo (Figure 6E) was also analyzed for each level. Fos-immunoreactive (-ir) nuclear profiles were dotted in a separate Photoshop layer using the paintbrush tool, and the dots were then counted in Image J (National Institutes of Health, Bethesda, MD). A cell nucleus was counted as Fos-ir if there was any detectable level of Fos protein in the nucleus that was above background levels (see arrowheads in Figure 7B for examples of Fos-ir cells).

In a first round of analyses, group data were analyzed by ANOVA followed by Fisher's PLSD, and simple regressions were used to determine correlations between Fos-ir cell counts and aggression displayed by dominant subjects, and aggression received by subordinate subjects. This was conducted separately for each sampling area (36 total sampling areas per side, distributed across 3 levels; Figure 6). Based on similar, statistically significant response properties in contiguous sampling areas, including those areas that are rostrocaudally contiguous (i.e., across levels, as determined by superimposition of photomicrographs), the 36 separate sampling areas shown in Figure 6 for a given side (i.e. left versus right) were reduced to the 9 functional zones shown in Figure 8. Although this first stage of analysis required many ANOVAs and regressions, we did not correct for multiple comparisons, as our goal was simply to reduce the large number of sampling areas to a smaller number of functionally comparable zones, in which case type I errors could lead to unnecessary “splitting” of zones. The data reported here are thus ANOVAs and regressions that were conducted for each of the 9 zones. Sampling areas were only pooled if absolutely all significant effects were shared. For instance, in the case that two adjacent sampling areas showed the same pattern of response in the group ANOVAs, but one area further exhibited a significant correlation between Fos-ir cell counts and behavior whereas the other area did not, we present these as separate zones in Figure 8 (e.g. zones 4 and 7; both zones were significantly activated in dominant waxbills in a resident intruder paradigm while zone 7 but not zone 4 was also activated in subordinate animals). In order to standardized data to the unit of Fos-ir cells/100 mm^2^, we summed the pixel areas for sampling boxes that comprise a given zone, divided this zone area by the pixel area of a 100 mm^2^ box, and then divided the total number of Fos-ir cells within that zone by the area of the zone.
